# Retraction of TREM2 aggravates sepsis by inhibiting fatty acid oxidation via the SHP1/BTK axis

**DOI:** 10.1172/JCI211875

**Published:** 2026-09-01

**Authors:** Siqi Ming, Xingyu Li, Qiang Xiao, Siying Qu, Qiaohua Wang, Qiongyan Fang, Pingping Liang, Yating Xu, Jingwen Yang, Yongqiang Yang, Xi Huang, Yongjian Wu

Original citation: *J Clin Invest.* 2025;135(1):e159400. https://doi.org/10.1172/JCI159400

Citation for this retraction: *J Clin Invest.* 2026;136(17):e211875. https://doi.org/10.1172/JCI211875

At the request of the corresponding author, the *JCI* is retracting this article. The authors recently became aware that in Figure 5F and 5G, the fatty acid oxidation (FAO) rates reported were incorrect due to a failure to normalize the data to the protein concentration in the lysates. After properly normalizing the FAO data, the authors found that treatment with ibrutinib or LFM-A13 did not rescue the FAO rate in TREM2-deficient macrophages. Given the importance of these figures in supporting the key findings of the paper, the authors felt that retraction was the most responsible course of action.

The authors apologize for the errors.

## Version 1 09/01/2026

Electronic publication

